# SSR-based molecular characterization of Verticillium wilt resistance in Iranian cotton cultivars

**DOI:** 10.1016/j.bbrep.2025.102059

**Published:** 2025-05-19

**Authors:** Sanaz Shahbazi, Sara Ghaffarian, Mohammad Razinataj, Mohammad Reza Zangi, Rasmieh Hamid, Bahman Panahi

**Affiliations:** a Department of Cellular and Molecular Biology, Faculty of Sciences, Azerbaijan Shahid Madani University, Tabriz, Iran; b Department of Plant Protection, Cotton Research Institute of Iran (CRII), Agricultural Research, Education and Extension Organization (AREEO), Gorgan, Iran; c Department of Plant Breeding, Cotton Research Institute of Iran (CRII), Agricultural Research, Education and Extension Organization (AREEO), Gorgan, Iran; d Department of Genomics, Branch for Northwest & West Region, Agricultural Biotechnology Research Institute of Iran (ABRII), Agricultural Research, Education and Extension Organization (AREEO), Tabriz, Iran

**Keywords:** Verticillium wilt, Cotton, Marker, PCoA, Resistance

## Abstract

Verticillium wilt (VW) is one of the most devastating diseases affecting cotton (*Gossypium* spp.), causing significant yield losses worldwide. The development of resistant cultivars is a primary strategy for managing this disease; however, conventional breeding approaches often encounter challenges in balancing resistance with high yield potential. This study aimed to assess the genetic diversity of 25 cotton cultivars using simple sequence repeat (SSR) markers and to identify key polymorphic markers associated with VW resistance. A total of 16 SSR markers were utilized, of which five (DPL405, DPL752, DPL866, DPL890, and DPL0022) were polymorphic. The polymorphism information content (PIC) values ranged from 0 to 0.49, with DPL405, DPL866, and DPL890 being the most informative. Principal coordinates analysis (PCoA) demonstrated genetic differentiation between resistant and sensitive cultivars, with the first axis explaining 41.19 % of the total variation. Resistant cultivars such as Leader, Golestan, and Arya clustered distinctly from sensitive varieties, confirming the effectiveness of the selected markers in genetic differentiation. Despite the promising results, key limitations include the relatively low overall marker polymorphism and the limited number of SSRs used, which may constrain broader genomic coverage and resolution. Nonetheless, the findings provide valuable insights for cotton breeding programs and highlight the potential of SSR markers in supporting marker-assisted selection (MAS) for Verticillium wilt resistance.

## Highlights

• Evaluate the genetic diversity and Verticillium wilt resistance of 25 Iranian cotton cultivars using SSR markers.
• Identify five polymorphic SSR loci, with DPL405, DPL866, and DPL0022 significantly associated with resistance phenotypes.
• Apply Principal Coordinates Analysis (PCoA) and UPGMA clustering to genetically differentiate resistant and susceptible cultivars.

## 1 Introduction

Cotton (*Gossypium* spp.) is a globally important crop cultivated extensively for its natural fiber and valuable byproducts, including seed oil and protein. It plays a crucial role in the economies of over 75 countries and supports the livelihoods of nearly 100 million farming families worldwide [[1], [2], [3]]. Beyond its role in the textile industry, cottonseed also contributes to food and feed production, underscoring the crop's broad agricultural and industrial relevance [4,5]. However, cotton production is increasingly threatened by various biotic stresses, particularly soil-borne diseases that reduce both yield and fiber quality. Among these, Verticillium wilt (VW), caused by the soil-borne fungus *Verticillium dahliae*, is one of the most destructive and persistent diseases affecting cotton. This pathogen has a wide host range and can survive in the soil for over a decade, rendering it extremely difficult to eradicate [[6], [7], [8], [9]]. Infected plants exhibit characteristic symptoms such as wilting, chlorosis, defoliation, and stunted growth, which can lead to yield losses ranging from 10 % to 35 %, depending on cultivar susceptibility and disease severity [[10], [11], [12]]. Although breeding efforts have led to the development of partially resistant cultivars, most commercial varieties remain moderately to highly susceptible. Moreover, traditional breeding approaches are often constrained by trade-offs among resistance, yield potential, and fiber quality [13]. As a result, the integration of molecular tools with classical breeding strategies has become essential for accelerating resistance improvement.

To address these challenges, molecular breeding tools such as simple sequence repeat (SSR) markers have emerged as valuable resources. SSR markers are highly polymorphic, reproducible, co-dominant, and effective for assessing genetic diversity and tagging agronomically important traits [14,15]. In recent years, several studies have successfully linked SSR markers to resistance genes and QTLs for VW in cotton and other crops [16], reinforcing their utility in marker-assisted selection (MAS). Despite these advances, the application of SSR markers for Verticillium wilt resistance remains underexplored in Iranian cotton germplasm, highlighting a critical gap in region-specific breeding resources.

Moreover, few studies to date have systematically integrated controlled phenotypic screening with molecular marker analysis to validate resistance-associated alleles in local cultivars [17,18]. This study aims to evaluate the genetic diversity of 25 Iranian native and commercial cotton cultivars using SSR markers and to identify polymorphic loci associated with VW resistance. By integrating SSR-based genotyping with phenotypic evaluation under controlled *V. dahliae* inoculation, this research offers valuable molecular insights that can accelerate the development of resistant cultivars through marker-assisted selection (MAS).

## 2 Material and methods

### 2.1 Plant material

The seeds of 25 cotton varieties, including modern cotton cultivars and local cultivars, were placed in an incubator after sanding. All international varieties and 16 of the local varieties reached the germination stage. After germination, uniform seedlings were transplanted into plastic pots (20 cm diameter) filled with a sterilized mixture of soil, sand, and peat moss (2:1:1). At regular intervals, they were watered and fertilized. Monopotassium Phosphate (MPK) (3 g per liter) was used as a fertilizer to promote growth, flowering, and increased resistance to stress. It was applied every 14 days. Each cultivar was evaluated under controlled greenhouse conditions using a completely randomized design (CRD) with three biological replicates. Each replicate consisted of five pots per cultivar, with three plants per pot, resulting in a total of 15 plants per cultivar. To impose Verticillium wilt stress under controlled conditions, the seedlings were inoculated with a highly virulent isolate of *Verticillium dahliae* obtained from naturally infected cotton fields in Golestan Province, Iran. The fungal inoculum was prepared by culturing the pathogen on potato dextrose agar (PDA) medium, followed by harvesting conidia and adjusting the suspension to a concentration of 1 × 10^7^ conidia/mL. The inoculation was carried out using the root-dip method: 21-day-old seedlings were uprooted, their roots gently washed, and submerged in the conidial suspension for 30 min before transplanting back into sterilized soil. Disease development was monitored weekly for a period of six weeks post-inoculation. Resistance evaluation was based on a visual disease severity rating scale from 0 to 4 (0 = no symptoms; 4 = severe wilting and defoliation), and the average disease severity index (DSI) was calculated for each cultivar. Cultivars with a mean DSI <1.5 were classified as resistant, 1.5–2.5 as moderately resistant, and >2.5 as susceptible. These cultivars were selected based on their historical performance in Verticillium wilt–prone regions of Golestan Province, including prior natural field evaluations conducted under endemic disease pressure in areas such as Gorgan. Once the plants reached full growth and the mature green leaves were fully developed, several leaves were harvested from each plant and immediately stored at −80 °C for DNA extraction. Among the studied cultivars, Varamin and Golestan were used as a standard sensitive and tolerant local cultivar, respectively. Further details of studied cultivars are provided in Table 1.

**Table 1:** The details of studied cotton cultivars used for screening of VW diseases resistance using molecular markers.

Cultivar ID	Cultivar name	Type	Source
I1	Odisa	Modern cotton	India
I2	Carisma	Modern cotton	Türkiye
I3	PG	Modern cotton	Türkiye
I4	My	Modern cotton	Türkiye
I5	Varamin	Modern cotton	Tehran Province-Iran
I6	Lotus	Modern cotton	Türkiye
I7	Golestan	Modern cotton	Golestan Province-Iran
I8	Sajedi	Modern cotton	Golestan Province-Iran
I9	Leader	Modern cotton	Türkiye
L1	Ghozeh ghermez barg sabz Qom	Local	Qom Province-Iran
L2	Shahrood	Local	Semnan Province-Iran
L3	Roodkhor Mehriz	Local	Yazd Province-Iran
L4	Damghan	Local	Semnan Province-Iran
L5	Herat Mehriz	Local	Yazd Province-Iran
L6	Ardakan-2	Local	Yazd Province-Iran
L7	Qom	Local	Qom Province-Iran
L8	Sabzevar 60-2	Local	Razavi Khorasan Province-Iran
L9	Aria	Local	Golestan Province-Iran
L10	Garmsar	Local	Semnan Province-Iran
L11	Alyaf rangi	Local	Golestan Province-Iran
L12	Neyshaboor	Local	Razavi Khorasan Province-Iran
L13	Lasjerd	Local	Semnan Province-Iran
L14	Sabzevar 60-1	Local	Razavi Khorasan Province-Iran
L15	Marvast mehriz korkdar	Local	Yazd Province-Iran
L16	Varzaneh Jozagh Isfahan	Local	Isfahan Province-Iran
L17	Ghozeh ghermezeh Kashmar	Local	North Khorasan Province-Iran
L18	Mahallat	Local	Markazi Province-Iran
L19	Semnan Sorkhe	Local	Semnan Province-Iran

### 2.2 DNA extraction

The extraction of high-quality DNA with a high yield is a critical factor in plant genetic analysis. Consistent DNA quality across cultivars is essential to enable accurate molecular comparisons among multiple individuals [14]. For this purpose, DNA was isolated from leaf tissues using a modified cetyltrimethylammonium bromide (CTAB) extraction protocol [15]. For every 80 mg of tissue, 1000 μL of CTAB extraction buffer (containing 2 % hexadecyltrimethylammonium bromide, 1.4 M NaCl, 0.2 % β-mercaptoethanol, 20 mM EDTA, and 100 mM Tris-HCl, pH 8) preheated to 65 °C was added to the leaves, which had been crushed and powdered using liquid nitrogen in a mortar and pestle. The mixture was then homogenized and incubated at 65 °C for 60 min with intermittent mixing. To address the high levels of polyphenolic compounds, present in cotton tissues, 1000 μL of a phenol:chloroform:isoamyl alcohol solution (25:24:1, v/v) was added, followed by vortexing and centrifugation. The resulting supernatant was transferred to a fresh tube. These extraction steps were repeated as necessary until the upper aqueous phase became clear. Subsequently, 500 μL of chilled isopropanol was added to each tube, and the samples were incubated at −20 °C for 30 min. The samples were then centrifuged at 13,000 rpm for 5 min, and the supernatant was discarded. Following this, 500 μL of 70 % ethanol was added to each tube, and the samples were gently inverted once to remove residual salts and improve DNA purity. The tubes were centrifuged again at 13,000 rpm for 5 min. After discarding the ethanol, the tubes were inverted onto filter paper and left to air-dry at room temperature for 15 min. The quality of the extracted DNA was evaluated by electrophoretic separation on a 3 % agarose gel stained with ethidium bromide (1 μg/mL) and by NanoDrop spectrophotometry. The purified DNA was then stored at −20 °C [15,16].

### 2.3 SSR profiling

Sixteen SSR primer pairs linked to QTLs for Verticillium wilt (VW) resistance were used for polymerase chain reaction (PCR) analyses. Details of the SSR marker sequences are provided in Table 2. PCR reactions were performed using 4 μL of distilled water, 5 μL of Master Mix buffer, 0.25 μL each of the forward and reverse primers, and 0.5 μL of purified genomic DNA, in a total reaction volume of 10 μL. PCR amplification was carried out for 37 cycles, each consisting of 30 s at 95 °C (denaturation), 30 s at 48 °C (annealing), and 30 s at 72 °C (extension), using an Applied Biosystems thermal cycler (Thermo Fisher Scientific, Waltham, MA, USA). The final PCR products were visualized under UV light following electrophoresis on 3 % agarose gels stained with ethidium bromide (1 μg/mL) [16].

**Table 2:** **Sequences, allele frequency, and diversity indices for the applied SSR markers.** Summary of SSR markers, including their sequences, allele frequency, number of alleles per marker, and diversity indices such as polymorphism information content (PIC), effective number of alleles (Ne), and Shannon's information index (I).

SSR ID	Primer Sequence	QTL	Chr. No.	Annealing Temperature	Reference	Na	PIC	Ne	I	He
DPL431	F:5′-CTATCACCCTTCTCTAGTTGCGTT-3' R:3′-ATCGGGCTCACAAACATCA-5′	–	AD-10	52	[16]	3	0.3648	1.000	0.000	0.000
DPL253	F:5′-TCACTATCTCAAGACCACCTTCAA-3' R:3′-AGTTCAAAGGACTCACCTGATGAT-5′	–	AD-11	53	[16]	2	0.2112	1.000	0.000	0.000
DPL513	F:5′-AGACCCGGCTACTACATGTTATCTT-3' R:3′-ACATACAGATGCTTCACACAAACA-5′	–	AD-1	52	[16]	1	0.0768	1.121	0.219	0.108
DPL405	F:5′-GAGATCCATGCTAACGTCTTACAAA-3' R:3′-ATGGGAGGAGGGAGTGGAA-5′	–	AD-14	51	[16]	3	0.3648	1.953	0.681	0.488
DPL752	F:5′-CACATCACCTAATTACCATTGAAGC-3' R:3′-TATCGTGAATATGTATGTGCGTGG-5′	–	AD-01	54	[16]	4	0.3648	1.953	0.681	0.488
DPL901	F:5′-GATGTGGTTAGGTGAGAAAGCA-3' R:3′-CTTTCCAGCTGCAGGACT-5′	–	AD-03,14	54	[18]	1	0.2688	1.000	0.000	0.000
DPL866	F:5′-AGAGTCAACTTCGACGCCAA-3' R:3′-CTTGCTCACTTCGATATGCT-5′	–	AD-26,12	54	[18]	1	0.4352	1.993	0.692	0.498
DPL890	F:5′-ACAGCATTAGCAGGCACCTT-3' R:3′-TATGAACGATGTGCTAGCCG-5′	–	AD-26	52	[18]	3	0.32	1.800	0.637	0.444
DPL307	F:5′-CACATCACCTAATTACCATTGAAGC-3' R:3′-TATCGTGAATATGTATGTGCGTGG-5′	–	AD-06	53	[18]	1	0.0768	1.121	0.219	0.108
DPL490	F:5′-AGTATCGTCACTTGTCAAAGTCCA-3' R:3′-CTCATGCATGCTTATCACACATC-5′	–	AD-01	54	[18]	4	0.4928	1.800	0.637	0.444
GH527	F:5′-AGCTGGAGGATTTCAGCTTGATTC-3' R:3′-ATGCCAGTTAACTTACCACGTTGG-5′	qVW-A7-1	7A7	54	[18]	1	0.3648	1.428	0.477	0.300
CIR295	F:5′-ATCACGCCAAAGAAAC-3' R:3′-TGTGGAGGCGTAAACT-5′	qVW-D2-1	AD-14	54	[29]	1	0.0768	1.121	0.219	0.108
JESPR12	F:5′-CCTAGACATCTGATTTAGCCAC-3' R:3′-GAAGAAGAAGAATCCGACAG-5′	qVW-A7-1	7A7	50	[29]	1	0.4928	1.800	0.637	0.444
DC20067	F:5′-ATGCAAACCATAAACATCT-3' R:3′-TGGGTTTGTGTGCTATCT-5′	qVW-A5-1	AD19,05	45	[29]	1	0.4992	1.263	0.363	0.208
DPL0022	F:5′-GGTGGGTTCTTCTGCAGGTATATT-3' R:3′-CCCTTTCAATGCTAGAAAGAAGTTG-5′	qVW-A5-1	AD-05	54	[29]	4	0.4608	1.993	0.692	0.498
GH215	F:5′-TCGGATACCACTTGTTGGAAGCA-3' R:3′-GTGTTAGTTATAAAAAGAATAGCAG-5′	qVW-A1-1	AD-13	51	[18]	3	0.32	1.121	0.219	0.108

### 2.4 Data analysis

For the diversity analysis of the studied cultivars based on molecular data, each amplified band was scored as present (1) or absent (0). The resulting binary data matrix was used to construct similarity matrices. Jacquard's similarity coefficient was employed to compute pairwise genetic similarity values [16]. The similarity matrix was then used for cluster analysis and dendrogram construction using the unweighted pair-group method with arithmetic mean (UPGMA), implemented in MEGA version 4.0. In addition, Principal Coordinates Analysis (PCoA) was performed using GenAlEx software version 6.5. The mean expected heterozygosity (He), Shannon's information index (I), and number of effective alleles (Ne) were also calculated for each primer using GenAlEx version 6.5. The effective number of alleles (Ne) represents the discriminatory power of a marker system in distinguishing genotypes per primer. It was calculated using the formula:

$$Ne=\frac{1}{1-h}$$

where h denotes the expected heterozygosity. For calculation of Shannon index (I) following formula was harnessed.

I=−PilogP

Where, Pi represents the frequency of the i-th allele at a given locus, and P denotes the frequency of a specific band in a genotype.

Additionally, calculation of polymorphism information content (PIC) value was performed using GeneAlEx software version 6.5 using following formula:

PIC=2fi(1−fi)

where fi is the frequency of the amplified allele and 1 −fi is the frequency of null allele.

## 3 Results

### 3.1 Total alleles amplification

**Fig. 1:**
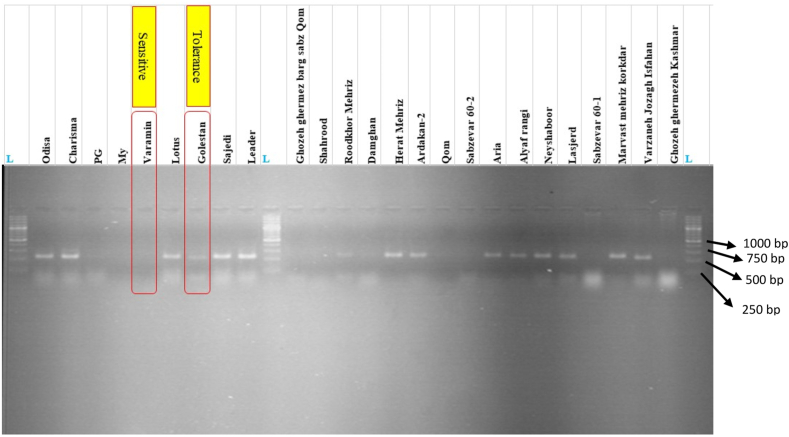
SSR profiling of local and international cultivars using DPL307, highlighting the discriminative efficiency of SSR markers for WV disease resistance. The banding patterns reveal genetic variation among cultivars, enabling differentiation between resistant and susceptible genotypes.

A total of 34 SSR alleles were amplified from the DNA of 25 cotton cultivars, demonstrating a moderate level of allelic diversity. The number of alleles detected per primer ranged from one to four, with an average of 2.13 alleles per SSR primer pair (Table 1). Among the primers tested, three SSR primers DPL752, DPL490, and DPL0022 exhibited the highest polymorphism, each producing four distinct alleles (See Fig. 1). In contrast, five primers, namely DPL431, DPL405, DPL890, GH215, and DPL253, generated two to three alleles per locus.

The remaining SSR markers, including DPL513, DPL901, DPL866, DPL307, GH527, CIR295, JESPR12, and DC20067, were monomorphic, producing only a single allele across all cultivars, indicating limited variation at these loci. Despite this, some monomorphic markers may still have utility for identifying specific genotypes or evaluating genetic stability in breeding lines.

The polymorphism information content (PIC) values, which measure the informativeness of a marker, ranged from 0.07 (CIR295, DPL513) to 0.499 (DC20067), with an average PIC value of 0.324 (Table 2). These results indicate that while some markers provided strong discriminatory power, others were less effective in differentiating genetic variation among the cultivars. This variation in PIC values reflects differences in allele number and frequency, highlighting the importance of selecting highly polymorphic markers for genetic diversity analyses in cotton breeding programs.

Moreover, the highest number of effective alleles (Ne = 1.993) was observed for DPL0022 and DPL866, followed closely by DPL405 and DPL752 (Ne = 1.953). In contrast, the lowest number of effective alleles (Ne = 1.000) was recorded for DPL431 and DPL253, indicating a lack of polymorphism at these loci (Table 2).

Shannon's information index (I), which provides an additional measure of genetic diversity, also varied among the markers. The lowest values were observed for DPL431, DPL253, and DPL901 (I = 0.0001), reflecting minimal allelic diversity. In contrast, DPL866 and DPL0022 exhibited the highest Shannon's index values (I = 0.692), suggesting a greater level of polymorphism and allelic richness among the studied cultivars. These findings highlight the varying discriminatory power of SSR markers and underscore their potential utility in assessing genetic diversity in cotton germplasm collections.

### 3.2 Principal Coordinates Analysis (PCoA)

Principal Coordinates Analysis (PCoA) is a multivariate technique used to visualize genetic similarities or differences within a dataset by projecting it into a lower-dimensional space. In the PCoA conducted for this study, eigenvalues were calculated for each axis, representing the proportion of total variance explained. The first axis had the highest eigenvalue (36.757), accounting for 41.19 % of the total variation, followed by the second axis (16.563) explaining 18.56 %, and the third axis (9.891) explaining 11.08 %.

**Fig. 2:**
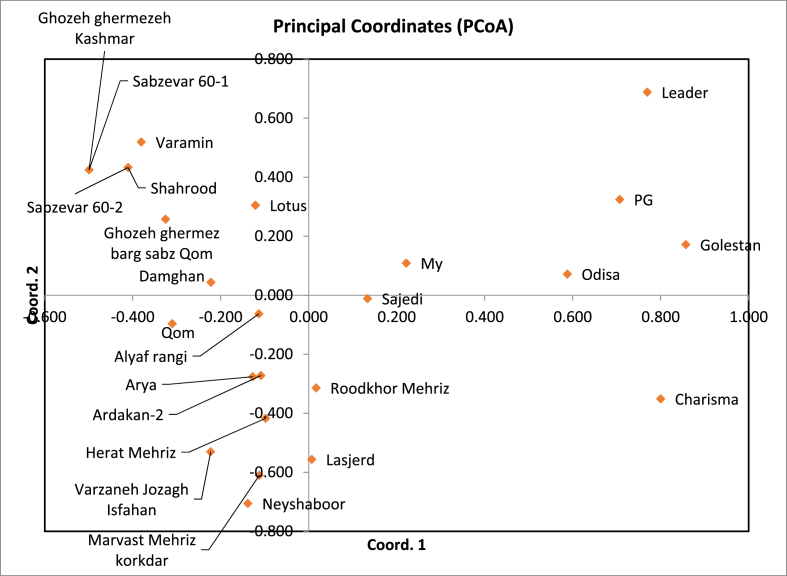
Principal Coordinate Analysis (PCO) of the studied cultivars based on SSR data. The plot illustrates the genetic relationships among cultivars, with the first two principal coordinates (PCo1 and PCo2) accounting for the major proportion of genetic variation. Cultivars are grouped according to their genetic similarity, distinguishing between WV-sensitive and WV-tolerant genotypes. The clustering pattern reflects the efficiency of SSR markers in differentiating between cultivars based on WV stress response.

The PCoA results revealed distinct genetic relationships among the studied cultivars, with a clear separation between resistant and susceptible genotypes. Specifically, resistant cultivars such as Leader, PG, Golestan, My, Odisa, Sajedi, Charisma, Roodkhor Mehriz, and Lasjerd clustered together on the right side of the PCoA plot. Within the resistant group, Odisa, Golestan, and PG exhibited high genetic similarity, suggesting close relatedness. In contrast, Charisma and Leader appeared genetically distant, despite both demonstrating resistance implying that different genetic mechanisms may underlie their resistance phenotypes. A substantial genetic distance was observed between Golestan (a resistant reference genotype) and Varamin (a susceptible control), reinforcing the validity of the phenotypic classification. Interestingly, My, a resistant cultivar, showed relative genetic similarity to Varamin, with a genetic distance of **5**. This unexpected proximity may reflect a shared ancestral background or the introgression of susceptible alleles into otherwise resistant genotypes. Additionally, the two susceptible cultivars, Varamin and Neyshaboor, exhibited genetic divergence, indicating heterogeneity within the susceptible group. Overall, the PCoA effectively distinguished resistant and susceptible cotton cultivars and highlighted genetically diverse sources of resistance, which may be valuable for breeding programs (see Fig. 2).

### 3.3 Hierarchical clustering

**Fig. 3:**
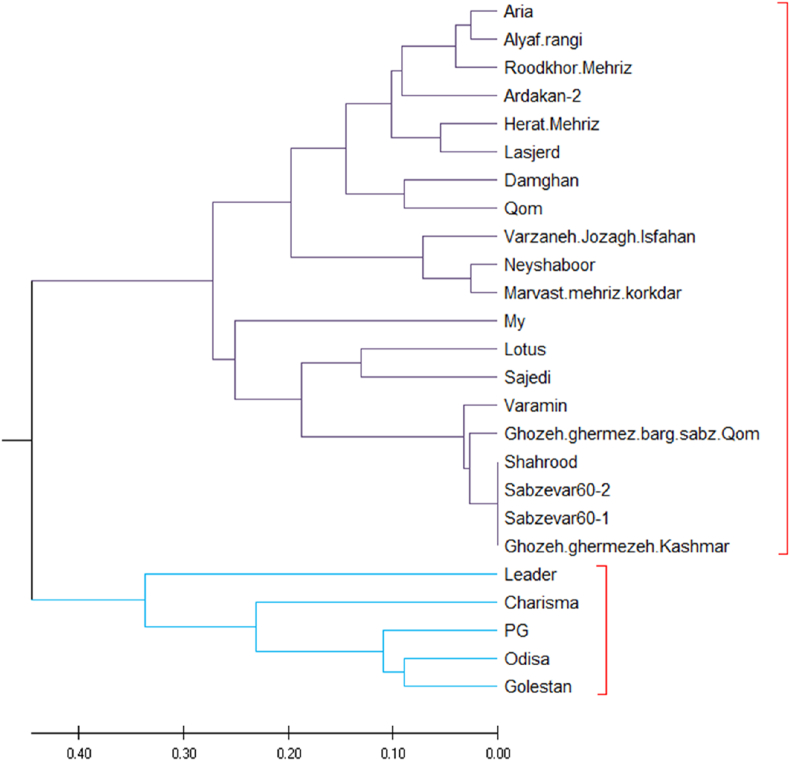
Dendrogram of hierarchical clustering of SSR data related to WV tolerance in 25 local and international cotton cultivars, constructed based on the Jaccard similarity coefficient using the Unweighted Pair Group Method with Arithmetic Mean (UPGMA). The dendrogram clusters cultivars into two major groups, distinguishing WV-tolerant and WV-sensitive genotypes.

A dendrogram based on UPGMA (Unweighted Pair Group Method with Arithmetic Mean) analysis using SSR marker data is shown in Fig. 3. The cultivars were divided into two different clusters that effectively distinguish between resistant and susceptible genotypes. Cluster I comprised predominantly VW-susceptible varieties, including several locally grown varieties as well as recognized susceptible standard varieties. It is noteworthy that varieties such as My and Lotus were classified in this cluster, which is consistent with previous phenotypic evaluations that identified them as VW susceptible. In contrast, Cluster II included VW-resistant genotypes such as Leader, Charisma, PG and Odisa, which are closely linked to Golestan, a well-established resistant standard. Overall, the UPGMA analysis provided valuable insights into the genetic structure of the investigated varieties and clearly distinguished between susceptible and resistant groups. This classification not only confirms the phenotypic data, but also supports the selection of diverse and robust parental lines for the development of VW-resistant cotton varieties.

## 4 Discussion

In this study, the genetic diversity of 25 cotton cultivars is analyzed using SSR markers, with a focus on distinguishing resistant and susceptible genotypes. SSR markers, commonly referred to as microsatellites, are abundant in genomic DNA and are ideal for assessing genetic variation and improving breeding efficiency [[18], [19], [20], [21], [22]]. Five markers DPL405, DPL752, DPL866, DPL890 and DPL0022 exhibited polymorphism. In genetics, a marker is called polymorphic if it has at least two alleles [[22], [23], [24], [25], [26]]. In particular, markers DPL405, DPL866 and DPL890 were the most informative and played a crucial role in detecting genetic variability and distinguishing between resistant and susceptible cultivars. Phenotypic screening under controlled V. *dahliae* inoculation allowed a robust classification of varieties based on Disease Severity Index (DSI) values. Cultivars with low DSI values were consistently clustered in the molecular analyses (e.g. PCoA and UPGMA dendrogram), showing that specific allele patterns at SSR loci, in particular DPL405, DPL866 and DPL0022, are associated with resistant phenotypes. This co-segregation of marker alleles with disease resistance suggests that these markers are either closely linked to or located within quantitative trait loci (QTLs) associated with Verticillium wilt resistance. Previous studies have also mapped these markers near resistance-associated loci. For example, DPL0022 and GH215 have been associated with QTLs on chromosomes A5 and A1, respectively, which are known to harbour VW resistance genes [16]. In our study, the presence of specific polymorphic alleles at these loci was consistent with phenotypic resistance responses, further confirming their potential as diagnostic markers. These results support the use of DPL405, DPL866 and DPL0022 in marker-assisted selection (MAS) for breeding VW resistance in cotton. The extent of polymorphism is also measured by heterozygosity, which ranged from 0.0 to 0.49 in this study [26]. This moderate heterozygosity reflects both informative variation and locus-specific conservation and makes these SSR markers suitable for differentiating resistance genotypes within elite germplasm pools. Consistent with our results, previous studies have identified specific SSR markers associated with QTLs for Verticillium wilt resistance, such as DPL0022 and GH215, which are associated with resistance on chromosomes A5 and A1, respectively [16].

In this study, markers such as DPL405, DPL866, DPL890, DPL0022 and JESPR12 showed higher Shannon index values. A higher Shannon index value indicates greater genetic variation and consequently a higher potential for genetic information, emphasizing the usefulness of these markers in cotton genetic studies [27].

Principal coordinate analysis (PCoA), a multidimensional scaling method for visualizing genetic similarities and differences, revealed that the first three principal axes accounted for most of the observed variance, with the first axis alone explaining 41.19 % of the variation [28]. In particular, resistant cultivars such as Leader, PG, Golestan, My, Odisa, Sajedi, Charisma, Roodkhor Mehriz and Lasjerd clustered clearly on the right side of the PCoA plot, confirming their genetic differentiation from susceptible cultivars. In agreement with our results, previous studies have further substantiated the potential of SSRs in differentiating Verticillium wilt-resistant cotton varieties. For example, Wang et al. [30] used a combination of AFLP and SSR markers to assess genetic diversity among Chinese G. *hirsutum* lines with known resistance or susceptibility to Fusarium and Verticillium wilts. Their analysis revealed considerable genetic differentiation among resistant genotypes, emphasizing the utility of SSRs in discriminating resistant lines. In addition, Jiang et al. [31] performed association mapping using SSR markers in a variety of cotton germplasm. They identified marker-trait associations for Verticillium wilt resistance, demonstrating the practical application of SSRs in association genetics and resistance screening. In addition, Bolek et al. [32] utilized microsatellites to reveal polymorphism between resistant and susceptible cotton parents. A large set of SSR primer pairs was used to map resistance genes. The clustering patterns observed in this study are consistent with previous results suggesting that resistant and susceptible cultivars form separate genetic groups [16]. The considerable genetic distance between the resistant control variety Golestan and the susceptible variety Varamin supports the reliability of these results. Remarkably, there is a relatively high genetic similarity between the resistant cultivar My and the susceptible cultivar Varamin. This indicates possible common genetic backgrounds or historical breeding influences and illustrates the complexity of the genomic diversity of cotton.

The identification of resistant varieties, particularly Arya, Sajedi, Charisma, PG, Golestan and Leader, provides valuable genetic resources for breeding programs to improve resistance to Verticillium wilt. Furthermore, the polymorphic markers identified in this study, in particular DPL405, DPL866 and DPL890, could be integrated into MAS programs for efficient and cost-effective screening of resistant genotypes.

Despite the successful identification of informative markers and characterization of genetic diversity, certain limitations should be considered. The relatively low number of polymorphic markers emphasizes the need for more extensive analyzes to capture greater genetic variability. In addition, environmental factors influencing Verticillium wilt resistance were not considered in this study. This should be considered in future studies to gain a more comprehensive understanding of resistance mechanisms. Further investigation of the genetic similarity between resistant and susceptible cultivars, such as My and Varamin, could also provide valuable insights into their genetic relationships.

## 5 Conclusion

This study offers valuable insights into the genetic diversity and Verticillium wilt (VW) resistance among 25 Iranian cotton cultivars using SSR markers. A total of five SSR markers were found to be polymorphic, with DPL405, DPL866, and DPL0022 showing strong associations with VW-resistant phenotypes. These markers demonstrated significant potential for distinguishing resistant from susceptible cultivars, underscoring their applicability in marker-assisted selection (MAS) for cotton breeding. Multivariate analyses, including UPGMA dendrogram and Principal Coordinates Analysis (PCoA), revealed distinct clustering patterns that effectively separated resistant and sensitive genotypes. Resistant cultivars such as Arya, Sajedi, Charisma, PG, Golestan, and Leader formed unique genetic groups, confirming the discriminatory power of these informative SSR markers. The observed co-segregation between molecular profiles and phenotypic responses under controlled inoculation reinforces the relevance of these markers for resistance screening and genetic differentiation in breeding programs. To expand on these findings, future research should incorporate a broader array of molecular markers particularly high-throughput platforms such as SNP arrays or genotyping-by-sequencing (GBS) to enhance genome-wide resolution. In addition, integrating environmental data and transcriptomic profiling may offer deeper insights into the molecular mechanisms underlying VW resistance. Further analysis of unexpected genetic similarities between certain resistant and susceptible cultivars, such as My and Varamin, could reveal cases of shared ancestry or historical introgression. Although the relatively low level of SSR polymorphism represents a limitation, this study provides a solid foundation for MAS-based strategies aimed at improving VW resistance in cotton and highlights the practical utility of SSR markers in resistance breeding.

## Author Contributions

BP conceived and designed the experiment. SS and SG performed the experiment and wrote the article. BP, SS, SG, RH, MZ, and MR contributed in reviewing the manuscript.

## Declaration of competing interest

Th authors declare that there is not any conflict of interest.
